# Environmental risk factors differ between rheumatoid arthritis with and without auto-antibodies against cyclic citrullinated peptides

**DOI:** 10.1186/ar2022

**Published:** 2006-07-27

**Authors:** Merete Pedersen, Søren Jacobsen, Mette Klarlund, Bo V Pedersen, Allan Wiik, Jan Wohlfahrt, Morten Frisch

**Affiliations:** 1 Department of Epidemiology Research, Danish Epidemiology Science Centre, Statens Serum Institut, Artillerivej 5, DK-2300 Copenhagen S, Denmark; 2 Department of Rheumatology, University Hospital of Copenhagen, Blegdamsvej 9, DK-2100 Copenhagen Ø, Denmark; 3 Department of Autoimmunology, Statens Serum Institut, Artillerivej 5, DK-2300, Copenhagen S, Denmark

## Abstract

The aim of this study was to evaluate new and previously hypothesised non-genetic risk factors for serologic subtypes of rheumatoid arthritis (RA) defined by the presence or absence of auto-antibodies to cyclic citrullinated peptides (CCP). In a national case-control study, we included 515 patients recently diagnosed with RA according to the American College of Rheumatology 1987 classification criteria and 769 gender- and age-matched population controls. Telephone interviews provided information about non-genetic exposures, and serum samples for patients were tested for anti-CCP-antibodies. Associations between exposure variables and risk of anti-CCP-positive and anti-CCP-negative RA were evaluated using logistic regression. A series of RA subtype-specific risk factors were identified. Tobacco smoking (odds ratio [OR] = 1.65; 95% confidence interval: 1.03–2.64, for >20 versus 0 pack-years) was selectively associated with risk of anti-CCP-positive RA, whereas alcohol consumption exhibited an inverse dose-response association with this RA subtype (OR = 1.98, 1.22–3.19, for 0 versus >0–5 drinks per week). Furthermore, coffee consumption (OR = 2.18; 1.07–4.42, for >10 versus 0 cups per day), ever use of oral contraceptives (OR = 1.65; 1.06–2.57) and having a first-degree relative with schizophrenia (OR = 4.18; 1.54–11.3) appeared more strongly associated with risk of anti-CCP-positive RA. Obesity was selectively associated with risk of anti-CCP-negative RA, with obese individuals being at more than 3-fold increased risk of this subtype compared with normal-weight individuals (OR = 3.45; 1.73–6.87). Age at menarche was the only examined factor that was significantly associated with both serologic subtypes of RA (p-trends = 0.01); women with menarche at age ≥ 15 years had about twice the risk of either RA subtype compared with women with menarche at age ≤ 12 years. Major differences in risk factor profiles suggest distinct etiologies for anti-CCP-positive and anti-CCP-negative RA.

## Introduction

A number of genetic and environmental factors have been implicated in the etiology of rheumatoid arthritis (RA). The only well-established environmental risk factor is tobacco smoking, which has been shown in a number of studies to be associated with increased RA risk [1-4]. Associations between RA and factors such as diet [5-7], coffee intake [8-10], alcohol [11-13], and body mass index [12-14] have also been studied, but the evidence to suggest a causal role of these factors is inconclusive. A widespread theory is that one or more infectious agents might act as initiator in the pathogenesis of RA by having antigens similar to host antigens, a mechanism referred to as molecular mimicry [15], but the evidence in favor of any particular microbe is weak. Because RA is approximately three times as common in women as in men, sex hormones and reproductive factors have been suggested as potentially involved in the etiology [16-18]. Furthermore, a sexually transmitted agent with a higher male-to-female than female-to-male transmission rate might theoretically explain the female predominance in RA, but only few studies have examined sexual behavior and venereal diseases as possible risk factors [19,20].

One possible explanation for conflicting results in etiologic studies might be that risk factors differ between subtypes of RA. It was recently demonstrated that smoking is selectively associated with rheumatoid factor (RF)-positive RA [21] or with RA positive for anti-cyclic citrullinated peptide (CCP) antibodies [22,23]. Also, coffee consumption has been found to be selectively associated with RF-positive RA, although the association diminished considerably after adjustment for tobacco smoking [9]. Further supporting the existence of etiologically distinct subtypes of RA, recent case-control studies have shown that measures of low socioeconomic status are predominantly associated with risk of RF-positive RA [24,25]. The aim of the present study was to evaluate both new and previously hypothesised non-genetic risk factors in serologically defined subgroups of anti-CCP-positive and anti-CCP-negative RA.

## Materials and methods

### Patients with RA and controls

The study was conducted as a frequency-matched case-control study. Patients with RA diagnosed within the previous 5 years were identified in rheumatology and internal medicine departments throughout Denmark, which has a predominantly Caucasian population of approximately 5.2 million inhabitants. To be included, patients had to be diagnosed with RA between ages 18 and 65 years and fulfill the American College of Rheumatology (ACR) 1987 classification criteria for RA [26] between August 1998 and July 2003. Information about date of diagnosis, defined as the date when the RA diagnosis was clinically confirmed by a rheumatologist, and cumulative fulfillment of the ACR 1987 classification criteria for RA was obtained from medical records by a rheumatologist at each department or by the project coordinator (MP) and a rheumatologist (MK) from the study team.

Controls who were frequency-matched by gender and birth year were randomly selected from the Danish population by means of the Civil Registration System, a national database that keeps track of all demographic changes in Denmark [27]. Using identical invitation letters to cases and controls, we aimed at a 1:1 case-control ratio for women and a 1:2 case-control ratio for men, but all invited subjects who agreed to participate were included. A higher number of controls per case among men was chosen in order to enhance statistical power in analyses of RA in men.

### Interview data

The questionnaire was tested in a pilot experiment comprising 50 patients with RA and 50 controls whose data are not included in this report. Three trained female medical students carried out all interviews between September 2002 and February 2004. Bimonthly meetings were held to ensure that all interviews were conducted in a uniform manner. Interviews were conducted as computer-assisted telephone interviews, and answers were entered directly into a database. Logical tests were built into the program to keep data entry errors at a minimum. Each telephone interview took approximately half an hour and included questions about a wide range of exposure and confounder variables, including level of education, age at menarche, parity, spontaneous abortions, breastfeeding, age at menopause, use of oral contraceptive pills and hormone replacement therapy, marital status, lifetime number of sexual partners of opposite sex, age at first sexual intercourse, lifetime number of anal-intercourse partners of opposite sex, lifetime number of same-sex sexual partners (male study participants only), lifetime number of prostitute visits (male study participants only), histories of venereal diseases (including chlamydia, genital herpes, acuminate condylomas, gonorrhea, and syphilis), smoker status, pack-years smoked (one pack-year equivalent to 20 cigarettes per day for 1 year with one cigarette equivalent to 1 g, one cheroot to 3 g, and one cigar to 4 g of tobacco), coffee, alcohol, and wine consumption 10 years before interview, frequency of fish intake as a hot meal or on bread (at least once a week, 1–3 times a month, less than once a month) 10 years before interview, intake of fish oil (ever/never), vegetarian diet (ever/never), body mass index at age 20 years and 10 years before interview, level of physical activity at work and during leisure time 10 years before interview, pets in childhood and in adulthood, histories of mononucleosis, hay fever, atopic dermatitis, asthma before age 45 years, stomach or duodenal ulcer, heavy diarrhea of at least 4 days' duration, type I diabetes, thyroid disease, periodontal disease, urinary tract infection, cancer, blood transfusion, tonsillectomy, adenoidectomy, appendectomy, splenectomy, and schizophrenia among first-degree relatives.

Blood samples were collected at rheumatology departments (patients) or by general practitioners (patients and controls), and serum was stored at -20°C. Anti-CCP immunoglobulin (Ig) G antibodies were determined by a second-generation enzyme-linked immunosorbent assay (ELISA) using the Immunoscan RA kit (Euro-Diagnostica AB, Malmö, Sweden), and parvovirus B19 IgG antibodies were determined by ELISA using the Biotrin Parvovirus B19 IgG kit (Dako Denmark A/S, Glostrup, Denmark) according to instructions provided by the manufacturers. Levels of IgG antibodies to Epstein-Barr viral capsid antigen (VCA) were determined in arbitrary units by ELISA using the Biotest anti-EBV VCA IgG kit (Meda A/S, Allerød, Denmark). A standard pool of serum was given the value of 100 arbitrary units. All samples were diluted 1:40 and tested together with dilutions (1:20, 1:40, 1:80, and 1:160) of the standard. Samples that were negative at 1:40 were retested undiluted together with dilutions of the standard. This enabled us to convert optical density values for the study samples into arbitrary units.

### Statistical analyses

To make exposure information comparable for patients and controls, a pseudo-year of diagnosis was attributed to controls according to the frequency distribution of year of RA diagnosis in patients of the same sex. Throughout, we disregarded information about exposures after the year (patients) or pseudo-year (controls) of diagnosis. We performed logistic regression analyses for men and women separately to study associations of exposure variables with overall RA risk regardless of RA subtype. For each exposure variable, we also combined information for women and men when there was no significant interaction with gender (likelihood ratio test, p > 0.05). In these analyses, we adjusted only for birth year, for year or pseudo-year of diagnosis, and (in combined analyses) for gender.

Risk factors for anti-CCP-positive and anti-CCP-negative RA were identified in a polytomous logistic regression model using these two groups of patients and the control group as the dependent variable. This model included gender, birth year, year or pseudo-year of diagnosis, place of residence (five categories), educational status (four categories), and exposure variables identified in a three-step selection process. (a) In the first step, exposure variables with a p value less than 0.05 in the gender-combined analyses described above were included in the final polytomous logistic regression model. (b) In the second step, we performed polytomous logistic regression to study the associations with risk of anti-CCP-positive and anti-CCP-negative RA for exposure variables with a p value less than 0.20 in the gender-combined analyses from the first step. The rather broad screening criterion of p less than 0.20 was used to ensure inclusion of potential RA subtype-specific risk factors for which associations might be blurred in subtype-unrestricted analyses. Adjustment was made for gender, birth year, and year or pseudo-year of RA diagnosis. Exposure variables with a p value less than 0.05 for at least one of the RA subtypes were included in the final multifactorial model. (c) In the third step, we considered a polytomous logistic regression model that included gender, birth year, year or pseudo-year of diagnosis, place of residence, educational status, and the exposure variables identified in steps one and two. Using the method of forward inclusion, we introduced in this model, one by one, all variables with a p value between 0.05 and 0.20 in at least one of the initial subtype-specific analyses in step two or the gender-combined analyses in step one. Introduced variables with a p value less than 0.05 for at least one RA subtype were included in the final model.

Tests for difference between exposure categories were evaluated by either trend tests or tests for homogeneity, as relevant. Trend tests were performed by treating categorical variables as continuous variables in the regression analyses. In trend tests for ordered categorical variables, representing an underlying continuous variable (for example, coffee consumption), categories were assigned the category median of the underlying variable. In trend tests for ordered categorical variables with no well-defined underlying continuous variable (for example, physical activity), the categories were assigned consecutive numbers starting with one. Tests for difference between risk factor associations with anti-CCP-positive and anti-CCP-negative RA were evaluated by polytomous logistic regression by comparing either subtype-specific trends or subtype-specific categorical variables, as relevant. All tests were likelihood-ratio-based. All analyses were carried out using SAS software (PROC LOGISTIC procedure in SAS 9.1; SAS Institute Inc., Cary, NC, USA). Throughout, we considered two-sided p values < 0.05 as statistically significant.

The study was approved by the Scientific Ethical Committees for Copenhagen and Frederiksberg (J. no. KF 01-039/01) and the Danish Data Protection Agency (2001-41-0658).

## Results

The study population consisted of 515 patients with RA (participation rate 83%) and 769 population controls (participation rate 64%). Selected socio-demographic characteristics are shown in Table 1. Patients had a mean disease duration of 2.3 years at inclusion in the study (range 0 to 5 years). No statistically significant differences were found between women and men for any of the exposures studied, so (whenever relevant) information was combined for women and men.

### Risk factors for RA overall

#### Reproductive factors

Reproductive factors were studied in women only (Table 2). Late age at menarche was associated with increased risk of RA (p-trend = 0.002), with women aged 15 years or older at menarche at almost double risk compared with those aged 12 years or less at menarche. There was no clear association with parity overall (odds ratio [OR] = 0.87; 95% confidence interval [CI]: 0.57 to 1.32, for parous versus nulliparous women) or with the specific number of children (p-trend = 0.15). Non-significant associations were also observed for spontaneous abortion, breastfeeding, age at menopause, ever use of oral contraceptives, and ever use of hormone replacement therapy (Table 2). Current use of oral contraceptives was also statistically unassociated with RA risk (OR = 1.00; 95% CI: 0.57 to 1.76, for current versus never use).

#### Marital status and sexual behaviour

Significantly more patients with RA than controls had remained unmarried (OR = 1.71; 95% CI: 1.14 to 2.58). Associations were not statistically significant for the lifetime number of sexual partners of opposite sex, age at first sexual intercourse, lifetime number of anal-intercourse partners of opposite sex, or history of venereal disease (Table 3). Also, among men, there was no association with ever having had homosexual experience (OR = 1.26; 95% CI: 0.28 to 5.77, three patients versus five controls) or with ever having visited a prostitute (OR = 0.95; 95% CI: 0.54 to 1.67, 24 patients versus 50 controls).

#### Smoking, coffee, alcohol, diet, body mass index, physical activity, and pets

Both former and current smokers were at significantly increased RA risk compared with never-smokers, and their risk increased proportionally with the number of pack-years smoked (Table 4). Coffee intake 10 years before interview was also positively associated with RA risk. Overall alcohol consumption 10 years before interview, and intake of wine specifically, exhibited an inverse dose-response association with RA risk. No statistically significant association was found with frequency of fish intake as a hot meal (p-trend = 0.17) or with intake of fish on bread, fish oil, or vegetarian diets (all p values > 0.3). Being obese 10 years before interview was marginally associated with increased RA risk. Furthermore, having a physically demanding job 10 years before interview was associated with significantly increased risk, whereas the opposite association was seen with level of physical activity during leisure time. No association was found between risk of RA and ever having had pets in the household as a child (p = 0.72), but pets in the household during adulthood was associated with reduced RA risk.

#### Viral antibodies in serum and self-reported health conditions

Seropositivity for parvovirus B19 antibodies (OR = 1.11; 95% CI: 0.81 to 1.52, 358 patients versus 409 controls) and above-median levels (that is, >44 arbitrary units) of IgG antibodies to viral capsid antigens of the Epstein-Barr virus (OR = 1.11; 95% CI: 0.86 to 1.44, 225 patients versus 250 controls) were both non-significantly associated with RA risk. Physician-verified asthma before age 45 years and a history of urinary tract infection were both associated with significantly reduced risk of RA. No statistically significant associations were seen with a range of other self-reported health conditions (Table 5).

### Risk factors for anti-CCP-positive and anti-CCP-negative RA

Serum samples were successfully analysed for anti-CCP antibodies for 445 (86%) of interviewed patients with RA. Of these, 309 (69%) were positive and 136 (31%) were negative for anti-CCP antibodies. Several statistically significant differences were seen between anti-CCP-positive and anti-CCP-negative RA (Table 6). Tobacco smoking and alcohol consumption were each selectively associated with risk of anti-CCP-positive RA, with tobacco smoking positively (p-trend = 0.03) and alcohol consumption inversely (p = 0.01) linked to risk of this RA subtype. Body mass index 10 years before interview was strongly and selectively associated with anti-CCP-negative RA (p-trend < 0.001), with obese (body mass index ≥30 kg/m^2^) individuals at more than threefold increased risk compared with normal-weight (body mass index 18.5 to <25 kg/m^2^) individuals (OR = 3.45; 95% CI: 1.73 to 6.87). Although other homogeneity tests for difference between risk associations with anti-CCP-positive and anti-CCP-negative RA were not statistically significant, ever use of oral contraceptives, a high intake of coffee, being unmarried or unemployed, and having a first-degree relative with schizophrenia were each more strongly associated with increased risk of anti-CCP-positive RA, whereas physician-verified asthma before age 45 years appeared to be more strongly inversely associated with anti-CCP-negative RA. Age at menarche was the only risk factor that was statistically significantly associated with both serologic subtypes of RA. Being 15 years or older at menarche was associated with double risk for both RA subtypes compared with having menarche at age 12 or younger (p-trend = 0.01 for both RA subtypes). Furthermore, having pets in adulthood was associated with decreased risk of both subtypes, although this association reached statistical significance for anti-CCP-positive RA only.

## Discussion

The present study provides strong support to recent proposals that RA may not be a single disease entity [28,29] but rather a clinical syndrome consisting of at least two distinct diseases with different etiologies. Recent observations suggest that smoking may be selectively associated with RF-positive RA [21] or anti-CCP-positive RA [22,23], notably in genetically predisposed individuals [22]. We confirm these previous findings that link tobacco smoking to an increased risk of anti-CCP-positive RA, but we also show that a range of other environmental risk factors differ between anti-CCP-positive and anti-CCP-negative RA.

Although unrelated to anti-CCP-negative RA, alcohol consumption 10 years before the interview was significantly inversely linked to risk of anti-CCP-positive RA, suggesting that alcohol may somehow protect against this RA subtype. Other observations are compatible with such a protective effect of alcohol. In one study, alcohol intake at the time of first visit to a rheumatology department was lower among women with RA than among women with soft tissue rheumatism or osteoarthritis [11], and other researchers have suggested a protective effect of alcohol that may be more pronounced in RF-positive than in RF-negative RA [12]. Possibly, the unspecific immune suppression exerted by alcohol [30] might somehow be beneficial in relation to risk of anti-CCP-positive RA if immune mechanisms involved in the pathogenesis of this RA subtype are affected.

Associations with coffee intake or use of oral contraceptive pills were not significantly different for the two subtypes of RA. However, after adjustment for tobacco smoking and a large number of other potential confounders, coffee and oral contraceptives appeared to be more strongly associated with risk of anti-CCP-positive RA than with anti-CCP-negative RA. Prior studies that did not make a clear distinction between anti-CCP-positive and anti-CCP-negative RA have yielded conflicting results for these exposures [8,9,31]. Additional studies are needed to determine whether coffee intake and use of oral contraceptives are subtype-specific risk factors associated with anti-CCP-positive RA only.

Although the test for difference between anti-CCP-positive and anti-CCP-negative RA was not significant, a strong positive association with having a first-degree relative with schizophrenia was present for anti-CCP-positive RA only. An association between schizophrenia and RA is potentially interesting because the prevalence of RA was recently found to be significantly higher in parents of schizophrenia patients compared with parents of non-psychiatric comparison subjects [32]. Furthermore, a genetic link between schizophrenia and RA has been suggested [33]. In apparent contrast, however, studies of intra-individual disease correlations have shown a *deficit *of RA diagnoses in patients with schizophrenia. If true, such an inverse association might suggest that predisposition to schizophrenia may somehow reduce the likelihood that the same individual will also develop RA. Of note, however, underdiagnosis of non-psychiatric health conditions in patients with schizophrenia may make intra-individual disease associations in schizophrenics hard to interpret [34]. The strength of the association observed here between anti-CCP-positive RA and schizophrenia in first-degree relatives is unlikely to be the result of spurious recall problems among study participants, but speculations about possible etiological implications should await confirmatory findings in other settings.

A high body mass index was strongly and selectively associated with increased risk of anti-CCP-negative RA, a finding that has not been reported previously. A chance finding appears unlikely given the highly significant trend with increasing body mass index and its specificity to anti-CCP-negative RA. Theoretically, however, although we included only patients who fulfilled ACR 1987 diagnostic criteria for RA as cases in our study, we cannot exclude the possibility that some anti-CCP-negative patients actually had inflammatory osteoarthritis, which is positively associated with body mass index. Consequently, confirmatory findings in other settings are needed. Possibly, the lacking identification of other etiological candidates for anti-CCP-negative RA might reflect that this RA subtype comprises a heterogeneous group of etiologically distinct inflammatory arthritides.

The female predominance in RA has prompted suggestions that sex hormones and reproductive factors may be etiologically involved [16-18]. However, in the present study, the only interesting reproductive factor was age at menarche, a finding that accords well with prior findings that women with early menarche are at comparatively low RA risk [35,36]. We also searched for clues to a possible venereal etiology, but associations with all examined sexual behaviors and sexually transmitted diseases were consistently non-significant. There was also no indication that infection with parvovirus B19 or Epstein-Barr virus, two previously suggested etiological candidates [37,38], would have any bearing on the risk of either anti-CCP-positive or anti-CCP-negative RA.

The patients in our study, identified at hospital departments of rheumatology and internal medicine throughout Denmark over a 5-year diagnostic period, are likely to be representative of patients with RA in need of hospital care. Our findings may not necessarily apply to milder cases of RA managed in outpatient settings, although we have little reason to believe that associations would differ in other RA populations. Although the participation rate was high (83%) among RA cases, invited population controls were slightly more reluctant to participate (64%). Theoretically, such a difference might lead to biased associations for some of the studied risk factors, to the extent these factors were also associated with the decision to accept or decline our invitation to participate. If invited subjects who did not want to participate comprised more tobacco smokers than those who actually participated, such non-random self-selection might have contributed to the observed positive dose-response association with tobacco consumption. However, the supporting evidence for a genuine RA subtype-specific effect of tobacco smoking in anti-CCP-positive RA which has been described by other researchers [22,23] and the lack of a spurious positive association between smoking and anti-CCP-negative RA in our study, suggest that the hypothetical impact, if any, of a relative deficit of tobacco-smoking controls would be small. Additionally, because tobacco and alcohol consumption are positively correlated behaviors, the observed inverse association between alcohol intake and risk of anti-CCP-positive RA cannot plausibly be explained by the lower participation rate among controls. If influenced at all, the inverse and RA subtype-specific association with alcohol consumption reported here is likely to be conservative.

We assessed risk factors retrospectively by means of telephone interviews, so the possible influence of recall problems among study participants needs consideration. Because patients with RA are unlikely to be aware of their anti-CCP antibody status and because exposures covered by our questionnaire are not broadly recognised as RA risk factors, we believe that misclassification arising from recall problems would most likely have been non-differential and tended to produce conservative ORs and blur risk factor differences between the two RA subtypes. The RA subtype-specific risk factor associations we observed are therefore unlikely to be the result of recall problems among our study participants.

## Conclusion

Our national case-control study addressed a large number of environmental factors potentially involved in the etiology of RA. Upon dichotomisation of patients with RA according to the presence or absence of anti-CCP-antibodies, we show that environmental risk factors differ considerably between anti-CCP-positive and anti-CCP-negative RA.

## Abbreviations

ACR = American College of Rheumatology; CCP = cyclic citrullinated peptide; CI = confidence interval; ELISA = enzyme-linked immunosorbent assay; Ig = immunoglobulin; OR = odds ratio; RA = rheumatoid arthritis; RF = rheumatoid factor; VCA = viral capsid antigen.

## Competing interests

The authors declare that they have no competing interests.

## Authors' contributions

MP participated in the study design, data collection, and drafting of the manuscript and performed statistical analyses. SJ and MF participated in the study design and drafting of the manuscript. MK participated in the data collection and drafting of the manuscript. BVP participated in the statistical analyses and drafting of the manuscript. AW participated in the study design and serum analyses and edited the manuscript. JW participated in study design, the statistical analyses, and the drafting of the manuscript. All authors read and approved the final version.

## Figures and Tables

**Table 1:** Demographic characteristics of 515 patients with rheumatoid arthritis and 769 population controls, Denmark 1998 to 2003

		**Patients with rheumatoid arthritis**	**Population controls**
			
**Mean age at diagnosis, years (range)^a^**		49 (18 to 65)	48 (16 to 68)
**Gender**	Women	366 (71.1%)	478 (62.2%)
	Men	149 (28.9%)	291 (37.8%)
			
**Birth year**	Before 1940	71 (13.8%)	98 (12.7%)
	1940 to 1949	171 (33.2%)	250 (32.5%)
	1950 to 1959	145 (28.2%)	223 (29.0%)
	1960 to 1969	77 (15.0%)	129 (16.8%)
	1970 or after	51 (9.9%)	69 (9.0%)
			
**Place of residence**	Copenhagen	86 (16.7%)	102 (13.3%)
	Suburbs of Copenhagen	90 (17.5%)	137 (17.8%)
	Other towns with ≥100,000 inhabitants	78 (15.2%)	77 (10.0%)
	Towns with 10,000 to 99,999 inhabitants	93 (18.1%)	197 (25.6%)
	Rural areas/towns with <10,000 inhabitants	168 (32.6%)	256 (33.3%)
			
**Education^b^**	No post-school education	123 (23.9%)	120 (15.6%)
	Semi-skilled worker, short education (<1 year) or apprentice	204 (39.6%)	293 (38.2%)
	Short or middle length advanced studies (1 to 4 years)	156 (30.3%)	265 (34.5%)
	Long advanced studies (>4 years)	32 (6.2%)	90 (11.7%)

^a^Age in controls calculated as pseudo-year of diagnosis minus birth year. ^b^One control had missing information about education.

**Table 2:** Reproductive factors in women and risk of rheumatoid arthritis, Denmark 1998 to 2003

	**Number of patients/controls**	**OR^a ^(95% CI)**
**Age at menarche**		
		
≤12 years	85/145	1 (ref)
13 years	84/130	1.08 (0.73 to 1.61)
14 years	88/105	1.39 (0.93 to 2.08)
≥15 years	94/80	1.87 (1.23 to 2.85)
Trend test (p)		0.002
		
**Number of live-born children**		
0	67/86	1 (ref)
1	81/85	1.10 (0.67 to 1.79)
2	133/196	0.76 (0.48 to 1.21)
3	63/84	0.81 (0.48 to 1.37)
≥4	21/27	0.73 (0.36 to 1.51)
Trend test (p)		0.15
		
**Spontaneous abortion**		
Never	237/292	1 (ref)
Ever	78/120	0.83 (0.59 to 1.18)
Test for homogeneity (p)		0.29
		
**Breastfeeding^b^**		
Never	19/18	1 (ref)
Ever	279/374	0.70 (0.35 to 1.39)
Test for homogeneity (p)		0.30
		
**Age at menopause^c^**		
<50 years	37/34	1 (ref)
≥50 years	47/49	0.87 (0.46 to 1.65)
Test for homogeneity (p)		0.68
		
**Oral contraceptive pills**		
Never	116/165	1 (ref)
Ever	249/312	1.24 (0.91 to 1.71)
Test for homogeneity (p)		0.18
		
**Hormone replacement therapy^d^**		
Never	328/436	1 (ref)
Ever	36/37	1.08 (0.63 to 1.86)
Test for homogeneity (p)		0.78

Due to missing values for some individuals, numbers do not always add up to 366 patients and 478 controls. ^a^Adjusted for birth year and year or pseudo-year of RA diagnosis. ^b^Among women with at least one child. ^c^Among postmenopausal women without histories of hormone replacement therapy or surgical removal of the uterus or ovaries. ^d^Among women age 45 years or older. CI, confidence interval; OR, odds ratio; ref, reference.

**Table 3:** Marital status, sexual behavior, and venereal diseases and risk of rheumatoid arthritis, Denmark 1998 to 2003

	**Women**		**Men**		**Both genders**
			
	**Number of patients/controls**	**OR^a ^(95% CI)**	**Number of patients/controls**	**OR^a ^(95% CI)**	**OR^a ^(95% CI)**
**Marital status**					
Married or cohabiting with partner	267/352	1 (ref)	116/229	1 (ref)	1 (ref)
Widowed	12/20	0.65 (0.29 to 1.45)	4/4	2.04 (0.44 to 9.51)	0.91 (0.46 to 1.81)
Divorced or separated	40/58	0.84 (0.54 to 1.33)	10/41	0.43 (0.20 to 0.92)	0.73 (0.50 to 1.06)
Unmarried	47/48	1.52 (0.93 to 2.49)	19/17	2.55 (1.16 to 5.61)	1.71 (1.14 to 2.58)
Test for homogeneity (p)		0.19		0.007	0.02
					
**Lifetime number of sexual partners of opposite sex**					
0 to 1	70/85	1 (ref)	16/44	1 (ref)	1 (ref)
2 to 5	160/184	1.13 (0.76 to 1.68)	51/79	1.74 (0.84 to 3.59)	1.26 (0.90 to 1.77)
6 to 10	79/124	0.87 (0.56 to 1.37)	36/58	1.62 (0.75 to 3.51)	1.04 (0.71 to 1.51)
11 to 20	35/63	0.69 (0.39 to 1.22)	21/48	1.26 (0.54 to 2.95)	0.84 (0.53 to 1.31)
21 to 30	8/9	1.15 (0.40 to 3.29)	6/32	0.48 (0.16 to 1.48)	0.65 (0.32 to 1.30)
>30	8/5	2.10 (0.62 to 7.16)	10/20	1.16 (0.38 to 3.55)	1.29 (0.63 to 2.64)
Trend test (p)		0.95		0.21	0.44
					
**Age at first sexual intercourse**					
≤14 years	24/36	0.98 (0.54 to 1.75)	11/27	0.69 (0.30 to 1.57)	0.88 (0.55 to 1.40)
15 to 17 years	170/228	1 (ref)	59/104	1 (ref)	1 (ref)
18 to 20 years	136/164	1.06 (0.77 to 1.46)	51/113	0.83 (0.51 to 1.37)	0.99 (0.76 to 1.29)
>20 years	29/42	0.80 (0.46 to 1.39)	26/44	1.11 (0.59 to 2.09)	0.95 (0.64 to 1.43)
Trend test (p)		0.62		0.53	0.98
					
**Lifetime number of anal-intercourse partners of opposite sex**					
0	273/339	1 (ref)	111/230	1 (ref)	1 (ref)
1	69/93	0.99 (0.68 to 1.45)	20/34	1.28 (0.67 to 2.42)	1.06 (0.77 to 1.46)
≥2	17/37	0.65 (0.34 to 1.22)	14/25	0.99 (0.45 to 2.20)	0.82 (0.51 to 1.32)
Trend test (p)		0.30		0.76	0.63
					
**Venereal disease^b^**					
Never	292/362	1 (ref)	117/223	1 (ref)	1 (ref)
Ever	67/102	0.85 (0.59 to 1.22)	30/57	1.02 (0.60 to 1.73)	0.90 (0.67 to 1.21)
Test for homogeneity (p)		0.38		0.95	0.48

Due to missing values for some individuals, numbers do not always add up to 366 female and 149 male patients with RA and 478 female and 291 male controls. ^a^Adjusted for birth year, year or pseudo-year of RA diagnosis, and (when combined) gender. ^b^One or more of the following: chlamydia, herpes, acuminate condylomas, gonorrhea, or syphilis. CI, confidence interval; OR, odds ratio; ref, reference.

**Table 4:** Tobacco smoking, coffee and alcohol intake, body mass index, physical activity, and pets and risk of rheumatoid arthritis, Denmark 1998 to 2003

	**Women**		**Men**		**Both genders**
			
	**Number of patients/controls**	**OR^a ^(95% CI)**	**Number of patients/controls**	**OR^a ^(95% CI)**	**OR^a ^(95% CI)**
**Smoker status**					
Never	128/223	1 (ref)	26/75	1 (ref)	1 (ref)
Former	68/76	1.69 (1.12 to 2.55)	40/79	1.58 (0.84 to 2.97)	1.57 (1.13 to 2.19)
Current	170/179	1.84 (1.33 to 2.54)	83/137	1.89 (1.09 to 3.30)	1.80 (1.37 to 2.36)
Test for homogeneity (p)		<0.001		0.08	< 0.001
					
**Pack-years smoked^b^**					
0 pack-years	128/223	1 (ref)	26/75	1 (ref)	1 (ref)
>0 to 10 pack-years	77/100	1.54 (1.04 to 2.28)	21/42	1.42 (0.69 to 2.91)	1.44 (1.02 to 2.02)
>10 to 20 pack-years	78/81	1.84 (1.23 to 2.77)	25/44	1.86 (0.92 to 3.77)	1.84 (1.30 to 2.59)
>20 pack-years	78/70	2.07 (1.35 to 3.16)	76/126	2.00 (1.12 to 3.58)	1.93 (1.40 to 2.68)
Trend test (p)		<0.001		0.03	<0.001
					
**Coffee consumption^c^**					
0 cups per day	70/116	1 (ref)	14/26	1 (ref)	1 (ref)
>0 to 5 cups per day	148/219	1.16 (0.76 to 1.79)	42/107	0.79 (0.35 to 1.81)	1.12 (0.77 to 1.64)
>5 to 10 cups per day	117/122	1.68 (1.05 to 2.70)	60/112	1.09 (0.49 to 2.46)	1.59 (1.07 to 2.38)
>10 cups per day	30/20	2.88 (1.43 to 5.79)	33/45	1.57 (0.64 to 3.84)	2.33 (1.40 to 3.87)
Trend test (p)		<0.001		0.05	<0.001
					
**Alcohol consumption^c,d^**					
0 drinks per week	79/80	1.36 (0.91 to 2.03)	11/13	1.38 (0.51 to 3.72)	1.34 (0.93 to 1.93)
>0 to 5 drinks per week	170/227	1 (ref)	42/75	1 (ref)	1 (ref)
>5 to 10 drinks per week	68/109	0.87 (0.60 to 1.27)	35/54	1.24 (0.68 to 2.26)	0.95 (0.70 to 1.30)
>10 to 15 drinks per week	29/34	1.21 (0.69 to 2.11)	11/41	0.42 (0.19 to 0.94)	0.80 (0.52 to 1.24)
>15 drinks per week	15/26	0.74 (0.37 to 1.47)	47/104	0.82 (0.47 to 1.43)	0.79 (0.53 to 1.17)
Trend test (p)		0.21		0.20	0.05
					
**Wine consumption^c^**					
0 glasses per week	120/135	1.23 (0.89 to 1.71)	36/65	0.89 (0.52 to 1.52)	1.09 (0.83 to 1.44)
>0 to 5 glasses per week	191/251	1 (ref)	87/139	1 (ref)	1 (ref)
>5 to 10 glasses per week	34/67	0.62 (0.39 to 1.00)	18/57	0.48 (0.26 to 0.89)	0.57 (0.39 to 0.83)
>10 to 15 glasses per week	11/16	0.82 (0.36 to 1.86)	2/17	0.15 (0.03 to 0.70)	0.53 (0.27 to 1.04)
>15 glasses per week	6/8	0.96 (0.31 to 2.92)	6/11	1.05 (0.35 to 3.09)	0.95 (0.44 to 2.02)
Trend test (p)		0.10		0.08	0.02
					
**Body mass index^c^**					
<18.5 kg/m^2 ^(underweight)	25/36	0.95 (0.52 to 1.73)	3/5	0.95 (0.19 to 4.72)	0.90 (0.52 to 1.56)
18.5 to < 25 kg/m^2 ^(normal weight)	232/327	1 (ref)	73/137	1 (ref)	1 (ref)
25 to < 30 kg/m^2 ^(overweight)	73/80	1.26 (0.87 to 1.85)	52/116	0.86 (0.54 to 1.37)	1.05 (0.79 to 1.40)
≥ 30 kg/m^2 ^(obese)	26/21	1.83 (0.97 to 3.44)	21/30	1.39 (0.71 to 2.71)	1.57 (1.01 to 2.44)
Trend test (p)		0.04		0.57	0.07
					
**Body mass index at age 20**					
<18.5 kg/m^2 ^(underweight)	56/62	1.39 (0.92 to 2.10)	4/9	0.75 (0.21 to 2.73)	1.29 (0.88 to 1.89)
18.5 to <25 kg/m^2 ^(normal weight)	258/371	1 (ref)	108/220	1 (ref)	1 (ref)
25 to <30 kg/m^2 ^(overweight)	36/30	1.72 (1.01 to 2.92)	28/38	1.65 (0.93 to 2.93)	1.61 (1.10 to 2.34)
≥ 30 kg/m^2 ^(obese)	4/6	1.33 (0.35 to 5.09)	5/5	1.92 (0.50 to 7.38)	1.59 (0.63 to 4.00)
Trend test (p)		0.64		0.05	0.15
					
**Physical activity at work^c^**					
Not physically demanding	46/94	1 (ref)	23/59	1 (ref)	1 (ref)
Slightly physically demanding	80/104	1.53 (0.95 to 2.47)	37/73	1.25 (0.65 to 2.41)	1.47 (1.01 to 2.14)
Moderately physically demanding	91/106	1.70 (1.07 to 2.72)	47/85	1.41 (0.75 to 2.67)	1.64 (1.13 to 2.37)
Very physically demanding	85/94	1.84 (1.14 to 2.96)	35/58	1.62 (0.82 to 3.18)	1.73 (1.18 to 2.53)
Unemployed	63/80	1.68 (0.95 to 2.98)	7/16	0.94 (0.30 to 2.93)	1.48 (0.91 to 2.42)
Trend test (p)^e,f^		0.01		0.11	0.005
					
**Physical activity in leisure time^c^**					
Not physically demanding	28/28	1 (ref)	25/29	1 (ref)	1 (ref)
Slightly physically demanding	125/150	0.80 (0.44 to 1.45)	52/76	0.84 (0.42 to 1.66)	0.77 (0.50 to 1.19)
Moderately physically demanding	180/260	0.65 (0.37 to 1.16)	59/145	0.44 (0.23 to 0.84)	0.56 (0.37 to 0.86)
Very physically demanding	33/39	0.92 (0.44 to 1.94)	13/41	0.26 (0.10 to 0.65)	0.61 (0.35 to 1.05)
Trend test (p)^e^		0.35		<0.001	0.004
					
**Pets as adult**					
Never	68/51	1 (ref)	22/36	1 (ref)	1 (ref)
Ever	298/427	0.46 (0.30 to 0.71)	127/255	0.85 (0.46 to 1.58)	0.56 (0.40 to 0.79)
Test for homogeneity (p)		<0.001		0.61	<0.001

Due to missing values for some individuals, numbers do not always add up to 366 female and 149 male patients with RA and 478 female and 291 male controls. ^a^Adjusted for birth year, year or pseudo-year of RA diagnosis, and (when combined) gender. ^b^One pack-year is equivalent to 7,300 cigarettes (20 cigarettes per day for 1 year). ^c^Ten years prior to interview. ^d^One drink is equivalent to one bottle of normal beer, 0.67 bottle of strong beer, or one glass of wine, dessert wine, or spirits. ^e^Trend tests on the original categories, which were enumerated by consecutive numbers starting with one. ^f^Including only individuals who had a job. CI, confidence interval; OR, odds ratio; ref, reference.

**Table 5:** Self-reported health conditions and risk of rheumatoid arthritis, Denmark 1998 to 2003

	**Women**		**Men**		**Both genders**
			
	**Number of patients/controls**	**OR^a ^(95% CI)**	**Number of patients/controls**	**OR^a ^(95% CI)**	**OR^a ^(95% CI)**
**Mononucleosis**	12/24	0.74 (0.35 to 1.56)	3/12	0.48 (0.13 to 1.84)	0.63 (0.33 to 1.20)^b^
**Hay fever^c^**	61/63	1.32 (0.89 to 1.97)	14/36	0.56 (0.27 to 1.17)	1.13 (0.81 to 1.58)
**Atopic dermatitis**	19/24	0.99 (0.51 to 1.90)	7/10	1.46 (0.51 to 4.20)	1.12 (0.65 to 1.94)
**Asthma^c,d^**	24/40	0.74 (0.43 to 1.28)	4/28	0.22 (0.07 to 0.67)	0.58 (0.36 to 0.93)^e^
**Stomach or duodenal ulcer**	20/32	0.70 (0.38 to 1.27)	12/30	0.70 (0.33 to 1.47)	0.73 (0.47 to 1.16)^b^
**Diarrhea**	49/84	0.72 (0.48 to 1.07)	27/53	1.01 (0.58 to 1.75)	0.81 (0.59 to 1.12)
**Type I diabetes^c^**	2/1	2.84 (0.22 to 36.05)	3/6	1.15 (0.26 to 5.00)	1.30 (0.39 to 4.34)
**Thyroid disease^c^**	38/34	1.45 (0.87 to 2.40)	3/2	2.32 (0.35 to 15.44)	1.52 (0.94 to 2.46)^b^
**Periodontal disease**	83/118	0.95 (0.67 to 1.33)	41/88	0.92 (0.57 to 1.47)	0.91 (0.70 to 1.19)
**Urinary tract infection**	171/256	0.76 (0.57 to 1.01)	13/40	0.57 (0.28 to 1.14)	0.73 (0.57 to 0.95)^e^
**Cancer**	12/19	0.70 (0.33 to 1.49)	4/2	5.51 (0.89 to 34.19)	1.01 (0.51 to 1.99)
**Blood transfusion**	51/66	0.99 (0.65 to 1.51)	12/26	0.84 (0.39 to 1.79)	0.98 (0.68 to 1.40)
**Tonsillectomy, adenoidectomy, appendectomy, or splenectomy**	163/214	0.97 (0.73 to 1.29)	63/124	1.00 (0.65 to 1.55)	0.98 (0.78 to 1.24)
**Schizophrenia among first-degree relatives**	6/5	2.19 (0.62 to 7.77)	6/6	1.77 (0.51 to 6.11)	2.08 (0.88 to 4.93)^b^

Numbers of patients and controls refer to participants who ever had the health condition in question. ^a^ORs represent comparisons between 'ever' versus 'never' (= reference) for each presented disease, with adjustment for birth year, year or pseudo-year of RA diagnosis, and (when combined) gender. ^b^Test for homogeneity 0.05 < p < 0.20. ^c^Physician-verified diagnosis. ^d^Asthma before age 45 years. ^e^Test for homogeneity p < 0.05. CI, confidence interval; OR, odds ratio.

**Table 6:** Non-genetic factors and risk of anti-CCP-positive and anti-CCP-negative rheumatoid arthritis, Denmark 1998 to 2003

	**Anti-CCP-positive rheumatoid arthritis**			**Anti-CCP-negative rheumatoid arthritis**			**Test for homogeneity anti-CCP-positive versus anti-CCP-negative rheumatoid arthritis (p)**
			
	**Number of patients/controls**	**OR^a ^(95% CI)**	**OR^b ^(95% CI)**	**Number of patients/controls**	**OR^a ^(95% CI)**	**OR^b ^(95% CI)**	
**Age at menarche (women only)**							
≤12 years	48/145	1 (ref)	1 (ref)	27/145	1 (ref)	1 (ref)	
13 years	52/130	1.24 (0.77 to 1.99)	1.33 (0.79 to 2.26)	22/130	0.88 (0.47 to 1.66)	1.06 (0.52 to 2.14)	
14 years	46/105	1.35 (0.82 to 2.22)	1.54 (0.88 to 2.69)	26/105	1.24 (0.67 to 2.29)	1.86 (0.93 to 3.70)	0.68
≥15 years	52/80	1.91 (1.16 to 3.16)	2.07 (1.18 to 3.63)	27/80	1.75 (0.93 to 3.29)	2.27 (1.13 to 4.57)	
Trend test (p)		0.01	0.01		0.06	0.01	
							
**Oral contraceptive pills (women only)**							
Never	64/165	1 (ref)	1 (ref)	33/165	1 (ref)	1 (ref)	
Ever	145/312	1.37 (0.93 to 2.02)	1.65 (1.06 to 2.57)	70/312	1.20 (0.73 to 1.98)	1.19 (0.68 to 2.07)	0.34
Test for homogeneity (p)		0.11	0.03		0.48	0.53	
							
**Marital status**							
Married or cohabiting with partner	227/581	1 (ref)	1 (ref)	106/581	1 (ref)	1 (ref)	
Widowed	8/24	0.79 (0.33 to 1.89)	0.50 (0.19 to 1.34)	5/24	0.86 (0.30 to 2.47)	0.72 (0.23 to 2.24)	
Divorced or separated	30/99	0.76 (0.48 to 1.19)	0.62 (0.36 to 1.04)	11/99	0.58 (0.29 to 1.13)	0.48 (0.22 to 1.06)	0.77
Unmarried	44/65	1.98 (1.25 to 3.14)	1.65 (0.96 to 2.84)	14/65	1.47 (0.75 to 2.92)	1.19 (0.53 to 2.67)	
Test for homogeneity (p)		0.01	0.03		0.24	0.28	
							
**Smoker status**							
Never	84/298	1 (ref)	1 (ref)	51/298	1 (ref)	1 (ref)	
Former	60/155	1.53 (1.02 to 2.29)	1.57 (0.99 to 2.48)	33/155	1.41 (0.84 to 2.34)	1.35 (0.76 to 2.39)	0.03
Current	165/316	2.13 (1.54 to 2.95)	1.73 (1.17 to 2.56)	52/316	1.01 (0.65 to 1.57)	0.83 (0.49 to 1.39)	
Test for homogeneity (p)		<0.001	0.02		0.35	0.22	
							
**Pack-years smoked^c^**							
0 pack-years	84/298	1 (ref)	1 (ref)	51/298	1 (ref)	1 (ref)	
>0 to 10 pack-years	51/142	1.32 (0.86 to 2.01)	1.31 (0.81 to 2.12)	27/142	1.22 (0.71 to 2.08)	1.20 (0.66 to 2.17)	
>10 to 20 pack-years	73/125	2.47 (1.65 to 3.69)	2.41 (1.51 to 3.82)	19/125	0.84 (0.46 to 1.52)	0.72 (0.37 to 1.40)	0.08
>20 pack-years	100/196	2.25 (1.53 to 3.32)	1.65 (1.03 to 2.64)	37/196	1.31 (0.78 to 2.19)	1.00 (0.54 to 1.88)	
Trend test (p)		<0.001	0.03		0.47	0.76	
							
**Coffee consumption^d^**							
0 cups per day	44/142	1 (ref)	1 (ref)	25/142	1 (ref)	1 (ref)	
>0 to 5 cups per day	110/326	1.20 (0.76 to 1.91)	1.33 (0.77 to 2.30)	53/326	0.84 (0.47 to 1.53)	0.79 (0.41 to 1.52)	
>5 to 10 cups per day	112/234	1.83 (1.12 to 2.97)	1.70 (0.95 to 3.05)	45/234	1.07 (0.57 to 2.00)	0.94 (0.47 to 1.90)	0.46
>10 cups per day	43/65	2.75 (1.52 to 4.99)	2.18 (1.07 to 4.42)	13/65	1.38 (0.61 to 3.15)	1.23 (0.48 to 3.16)	
Trend test (p)		<0.001	0.02		0.22	0.44	
							
**Alcohol consumption^d,e^**							
0 drinks per week	58/93	1.68 (1.10 to 2.55)	1.98 (1.22 to 3.19)	18/93	0.88 (0.48 to 1.64)	0.72 (0.34 to 1.48)	
>0 to 5 drinks per week	124/302	1 (ref)	1 (ref)	60/302	1 (ref)	1 (ref)	
>5 to 10 drinks per week	63/163	0.96 (0.66 to 1.39)	1.10 (0.73 to 1.66)	27/163	0.89 (0.53 to 1.48)	1.01 (0.58 to 1.74)	0.01
>10 to 15 drinks per week	26/75	0.82 (0.49 to 1.38)	0.98 (0.55 to 1.76)	9/75	0.67 (0.31 to 1.45)	0.78 (0.33 to 1.84)	
>15 drinks per week	32/130	0.58 (0.35 to 0.94)	0.60 (0.35 to 1.04)	21/130	1.36 (0.73 to 2.52)	1.36 (0.68 to 2.74)	
Trend test (p)		0.002	0.01		0.45	0.34	
							
**Wine consumption^d^**							
0 glasses per week	96/200	1.18 (0.86 to 1.63)	1.18 (0.82 to 1.71)	36/200	0.98 (0.62 to 1.55)	0.86 (0.51 to 1.45)	
>0 to 5 glasses per week	168/390	1 (ref)	1 (ref)	72/390	1 (ref)	1 (ref)	
>5 to 10 glasses per week	30/124	0.52 (0.33 to 0.81)	0.59 (0.36 to 0.98)	17/124	0.77 (0.43 to 1.38)	0.76 (0.39 to 1.45)	0.03
>10 to 15 glasses per week	7/33	0.46 (0.20 to 1.09)	0.45 (0.17 to 1.21)	6/33	0.99 (0.38 to 2.56)	0.83 (0.27 to 2.61)	
>15 glasses per week	5/19	0.66 (0.24 to 1.84)	0.90 (0.29 to 2.76)	5/19	1.59 (0.54 to 4.66)	2.29 (0.71 to 7.42)	
Trend test (p)		0.003	0.04		0.77	0.46	
							
**Body mass index^d^**							
<18.5 kg/m^2 ^(underweight)	19/41	1.22 (0.65 to 2.29)	0.88 (0.43–1.80)	4/41	0.34 (0.10 to 1.19)	0.35 (0.09 to 1.34)	
18.5 to <25 kg/m^2 ^(normal weight)	184/464	1 (ref)	1 (ref)	77/464	1 (ref)	1 (ref)	
25 to <30 kg/m^2 ^(overweight)	77/196	1.04 (0.74 to 1.45)	1.01 (0.69 to 1.47)	35/196	1.20 (0.75 to 1.93)	1.24 (0.74 to 2.10)	0.004
≥30 kg/m^2 ^(obese)	25/51	1.34 (0.79 to 2.28)	1.15 (0.62 to 2.13)	20/51	3.22 (1.73 to 5.98)	3.45 (1.73 to 6.87)	
Trend test (p)		0.53	0.63		<0.001	<0.001	
							
**Physical activity at work^d^**							
Not physically demanding	34/153	1 (ref)	1 (ref)	23/153	1 (ref)	1 (ref)	
Slightly physically demanding	63/177	1.60 (0.99 to 2.58)	1.10 (0.65 to 1.87)	39/177	1.51 (0.85 to 2.69)	1.41 (0.74 to 2.67)	
Moderately physically demanding	93/191	2.24 (1.41 to 3.54)	1.64 (0.98 to 2.73)	30/191	1.08 (0.59 to 1.97)	1.02 (0.52 to 1.99)	0.84
Very physically demanding	70/152	2.03 (1.26 to 3.27)	1.18 (0.68 to 2.06)	34/152	1.53 (0.84 to 2.77)	1.33 (0.66 to 2.67)	
Unemployed	48/96	2.58 (1.45 to 4.61)	1.95 (1.00 to 3.81)	10/96	0.47 (0.18 to 1.23)	0.41 (0.13 to 1.23)	
Trend test (p)^f,g^		0.002	0.30		0.34	0.57	
							
**Physical activity in leisure time^d^**							
Not physically demanding	35/57	1 (ref)	1 (ref)	11/57	1 (ref)	1 (ref)	
Slightly physically demanding	108/226	0.74 (0.45 to 1.21)	0.97 (0.56 to 1.70)	49/226	0.95 (0.45 to 1.98)	0.87 (0.39 to 1.94)	
Moderately physically demanding	137/405	0.51 (0.32 to 0.83)	0.76 (0.43 to 1.32)	66/405	0.69 (0.33 to 1.42)	0.76 (0.34 to 1.67)	0.75
Very physically demanding	29/80	0.60 (0.32 to 1.13)	0.90 (0.44 to 1.86)	10/80	0.60 (0.23 to 1.59)	0.59 (0.20 to 1.70)	
Trend test (p)^f^		0.008	0.32		0.10	0.29	
							
**Pets as adult**							
Never	52/87	1 (ref)	1 (ref)	20/87	1 (ref)	1 (ref)	
Ever	257/682	0.59 (0.40 to 0.87)	0.62 (0.39 to 0.99)	116/682	0.65 (0.37 to 1.14)	0.61 (0.33 to 1.16)	0.98
Test for homogeneity (p)		0.01	0.04		0.13	0.13	
							
**Asthma^h^**							
Never	288/699	1 (ref)	1 (ref)	131/699	1 (ref)	1 (ref)	
Ever	21/68	0.73 (0.43 to 1.23)	0.58 (0.32 to 1.08)	4/68	0.32 (0.11 to 0.91)	0.28 (0.09 to 0.85)	0.23
Test for homogeneity (p)		0.23	0.09		0.03	0.02	
							
**Schizophrenia among first-degree relatives**							
No	299/758	1 (ref)	1 (ref)	134/758	1 (ref)	1 (ref)	
Yes	10/11	3.36 (1.32 to 8.51)	4.18 (1.54 to 11.3)	2/11	1.16 (0.24 to 5.55)	0.51 (0.06 to 4.44)	0.16
Test for homogeneity (p)		0.01	0.005		0.85	0.54	

Due to missing values for some individuals, numbers do not always add up to 309 anti-CCP-positive and 136 anti-CCP-negative patients with RA and 769 controls. ^a^Adjusted for gender, birth year, and year or pseudo-year of RA diagnosis. ^b^Adjusted for gender, birth year, year or pseudo-year of RA diagnosis, place of residence, educational status, history of urinary tract infection, and all other variables in the table. Smoking variables not adjusted for the other measure of smoking, and alcohol and wine variables not adjusted for each other. ^c^One pack-year is equivalent to 7,300 cigarettes (20 cigarettes per day for 1 year). ^d^Ten years prior to interview. ^e^One drink is equivalent to one bottle of normal beer, 0.67 bottle of strong beer, or one glass of wine, dessert wine, or spirits. ^f^Trend tests based on original categories enumerated by consecutive numbers starting with one. ^g^Including only individuals who were employed. ^h^Physician-verified diagnosis before age 45 years. CI, confidence interval; OR, odds ratio; ref, reference.
